# Supplementary material for: The Construction and Exploration of a Comprehensive MicroRNA Centered Regulatory Network in Foxtail Millet (Setaria italica L.)
Source: Front Plant Sci. 2022 May 6;13:848474. doi: 10.3389/fpls.2022.848474 (PMC9121102; doi:10.3389/fpls.2022.848474)
Supplement: Supplementary file 3 [file Data_Sheet_3.zip › Sit-miR393a_Seita.7G078300.1_2366_TPlot.pdf]

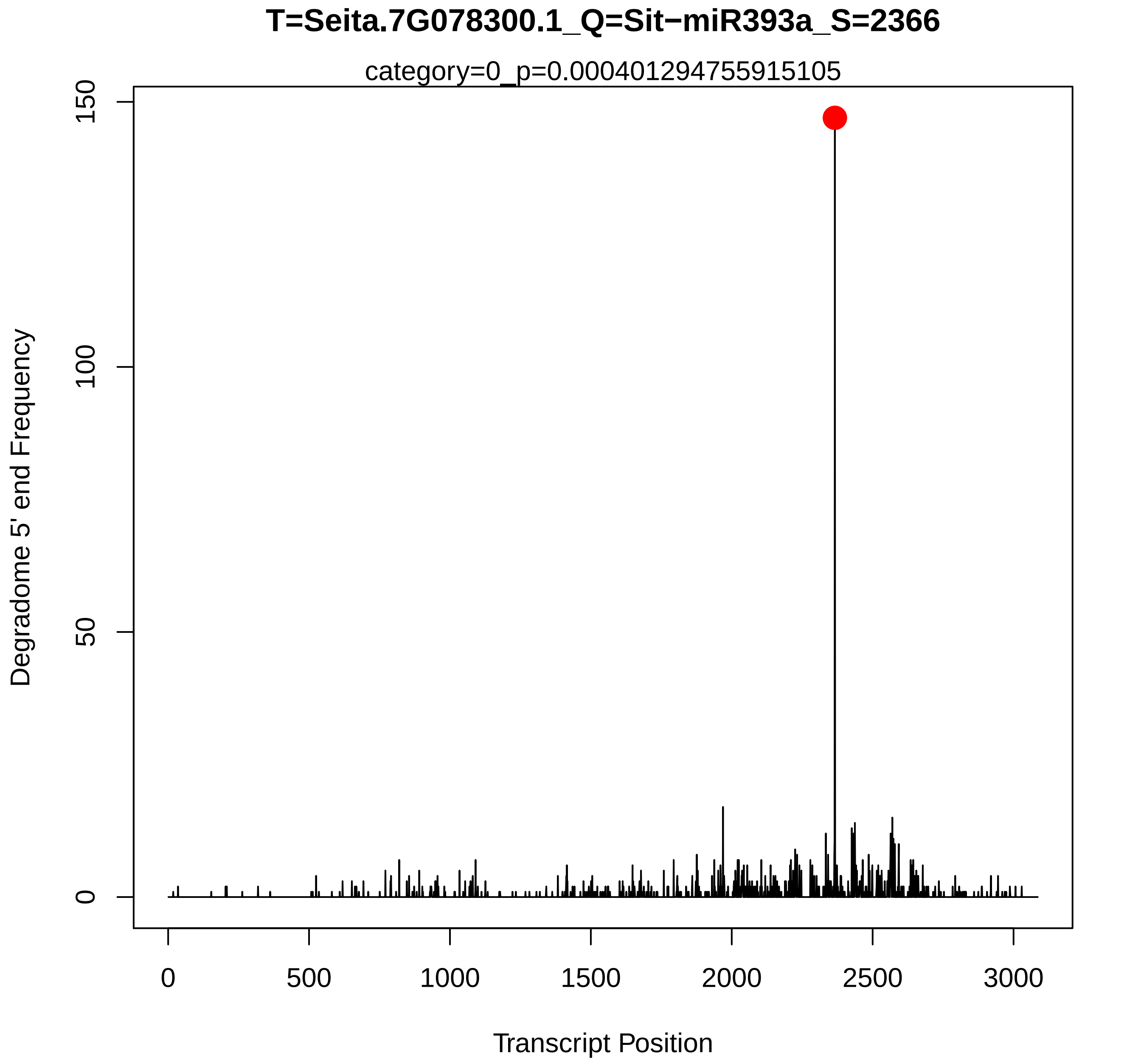

T=Seita.7G078300.1_Q=Sit−miR393a_S=2366
category=0_p=0.000401294755915105
Degradome 5' end Frequency
150
100
50
0
0
500
1000
1500
2000
2500
3000
Transcript Position
